# Imaging asparaginyl endopeptidase (AEP) in the live brain as a biomarker for Alzheimer’s disease

**DOI:** 10.1186/s12951-021-00988-0

**Published:** 2021-08-19

**Authors:** Shan-Shan Wang, Zi-Kai Liu, Jing-Jing Liu, Qing Cheng, Yan-Xia Wang, Yan Liu, Wen-Wen Ni, Hong-Zhuan Chen, Mingke Song

**Affiliations:** 1 Department of Pharmacology and Chemical Biology, Institute of Medical Sciences, Shanghai Jiao Tong University School of Medicine, 280 South Chongqing Road, Shanghai, 200025 China; 2 Institute of Interdisciplinary Integrative Biomedical Research, Shanghai University of Traditional Chinese Medicine, 1200 Cailun Road, Shanghai, 201210 China

**Keywords:** Alzheimer’s disease, Early diagnosis, Biomarker, Asparagine endopeptidase, Legumain, Live brain imaging, Fluorescent probe, Cy5.5, Gold nanoparticle, Click cycloaddition

## Abstract

**Background:**

Discovery of early-stage biomarkers is a long-sought goal of Alzheimer’s disease (AD) diagnosis. Age is the greatest risk factor for most AD and accumulating evidence suggests that age-dependent elevation of asparaginyl endopeptidase (AEP) in the brain may represent a new biological marker for predicting AD. However, this speculation remains to be explored with an appropriate assay method because mammalian AEP exists in many organs and the level of AEP in body fluid isn’t proportional to its concentration in brain parenchyma. To this end, we here modified gold nanoparticle (AuNPs) into an AEP-responsive imaging probe and choose transgenic APPswe/PS1dE9 (APP/PS1) mice as an animal model of AD. Our aim is to determine whether imaging of brain AEP can be used to predict AD pathology.

**Results:**

This AEP-responsive imaging probe AuNPs-Cy5.5-A&C consisted of two particles, AuNPs-Cy5.5-AK and AuNPs-Cy5.5-CABT, which were respectively modified with Ala–Ala–Asn–Cys–Lys (AK) and 2-cyano-6-aminobenzothiazole (CABT). We showed that AuNPs-Cy5.5-A&C could be selectively activated by AEP to aggregate and emit strong fluorescence. Moreover, AuNPs-Cy5.5-A&C displayed a general applicability in various cell lines and its florescence intensity correlated well with AEP activity in these cells. In the brain of APP/PS1 transgenic mice , AEP activity was increased at an early disease stage of AD that precedes formation of senile plaques and cognitive impairment. Pharmacological inhibition of AEP with δ-secretase inhibitor 11 (10 mg kg^−1^, p.o.) reduced production of β-amyloid (Aβ) and ameliorated memory loss. Therefore, elevation of AEP is an early sign of AD onset. Finally, we showed that live animal imaging with this AEP-responsive probe could monitor the up-regulated AEP in the brain of APP/PS1 mice.

**Conclusions:**

The current work provided a proof of concept that assessment of brain AEP activity by in vivo imaging assay is a potential biomarker for early diagnosis of AD.

**Graphical abstract:**

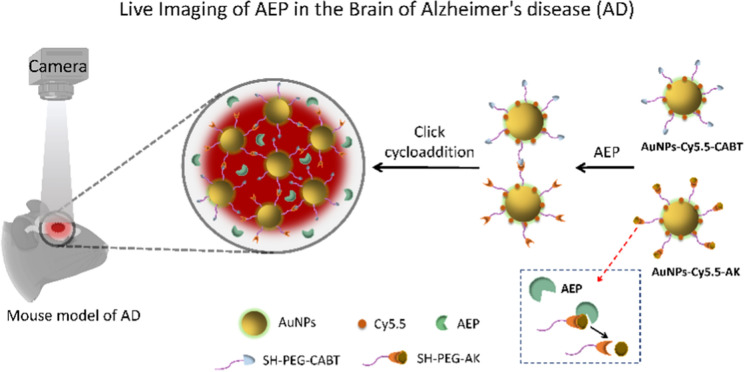

**Supplementary Information:**

The online version contains supplementary material available at 10.1186/s12951-021-00988-0.

## Introduction

Early diagnosis of Alzheimer’s disease (AD) remains a challenge [1–3]. β-amyloid (Aβ) plaques and tau neurofibrillary tangles are the primary pathological hallmarks of AD [4]. Currently established AD diagnostic biomarkers are based on concentration of amyloid-beta 42 (Aβ42) in cerebrospinal fluid (CSF), CSF total tau (T-tau) and phosphorylated tau (P-tau), and Aβ positron-emission tomography (PET) imaging [4, 5]. These biomarkers mainly mirror the endpoint of amyloid precursor protein (APP) metabolism and tauopathy. They are used to diagnose AD dementia (ADD) and forewarn the conversion from mild cognitive impairment (MCI) to ADD in patients, but hard to predict earlier stage of AD [4–6]. To meet the requirement of early AD diagnosis, new biomarkers need to be identified from the early disease stage, e.g., the upstream signals of amyloid cascade.

For most AD cases, age is the greatest risk factor as the incidence rises significantly with ageing [7, 8]. However, it is not clear how ageing promotes amyloidogenic APP processing until recently the lysosomal asparaginyl endopeptidase (AEP) or Legumain was identified as a critical link between aging and AD onset [9–12]. AEP was found elevated and activated in animal and human AD brains during ageing [12–14]. The elevated AEP acts as a δ-secretase to cut APP into amyloidogenic fragments, facilitating β-secretase and γ-secretase to cleave APP fragments and resulting in overproduction of Aβ [12, 13, 15]. Deletion or pharmacological inhibition of AEP prevented progression of Aβ-related pathologies in animal AD models [12, 15, 16]. Hence, AEP precedes β- and γ-secretase to process APP and plays a vital role in AD pathogenesis. These findings strongly suggest that age-dependent increase of AEP activity is a biological marker that reflects early pathological stage of AD. However, this tempting speculation needs to be explored at least through a preclinical study and using an appropriate measurement.

Mammalian AEP is widely distributed in kidney, liver, spleen, brain, and many tumor tissues [17, 18]. AEP can translocate from the lysosomal system to cytoplasm and the cell surface, sequentially into body fluid and circulation [17]. Levels of AEP in body fluid is complicated by different origins and not proportional to its concentration in brain parenchyma, therefore a fluid-based assay does not reflect brain AEP activity. In recent years, activity-based probes (ABPs) and imaging analysis had been employed to detect cellular AEP activity [19]. To date, several types of fluorescent imaging probes have been developed for detection of AEP, such as AEP inhibitor-dependent Cy5 fluorophore or quenched activity-based probes [20–25]. These fluorescent probes have an overall “turned on” property when activated by AEP but without evidence that their fluorescent intensity can correlate closely with changes of cellular AEP activity. Moreover, previously reported AEP imaging probes were mostly applied to tumor studies; none of them had ever been used to detect AEP activity in degenerative brain diseases.

**Scheme 1:**
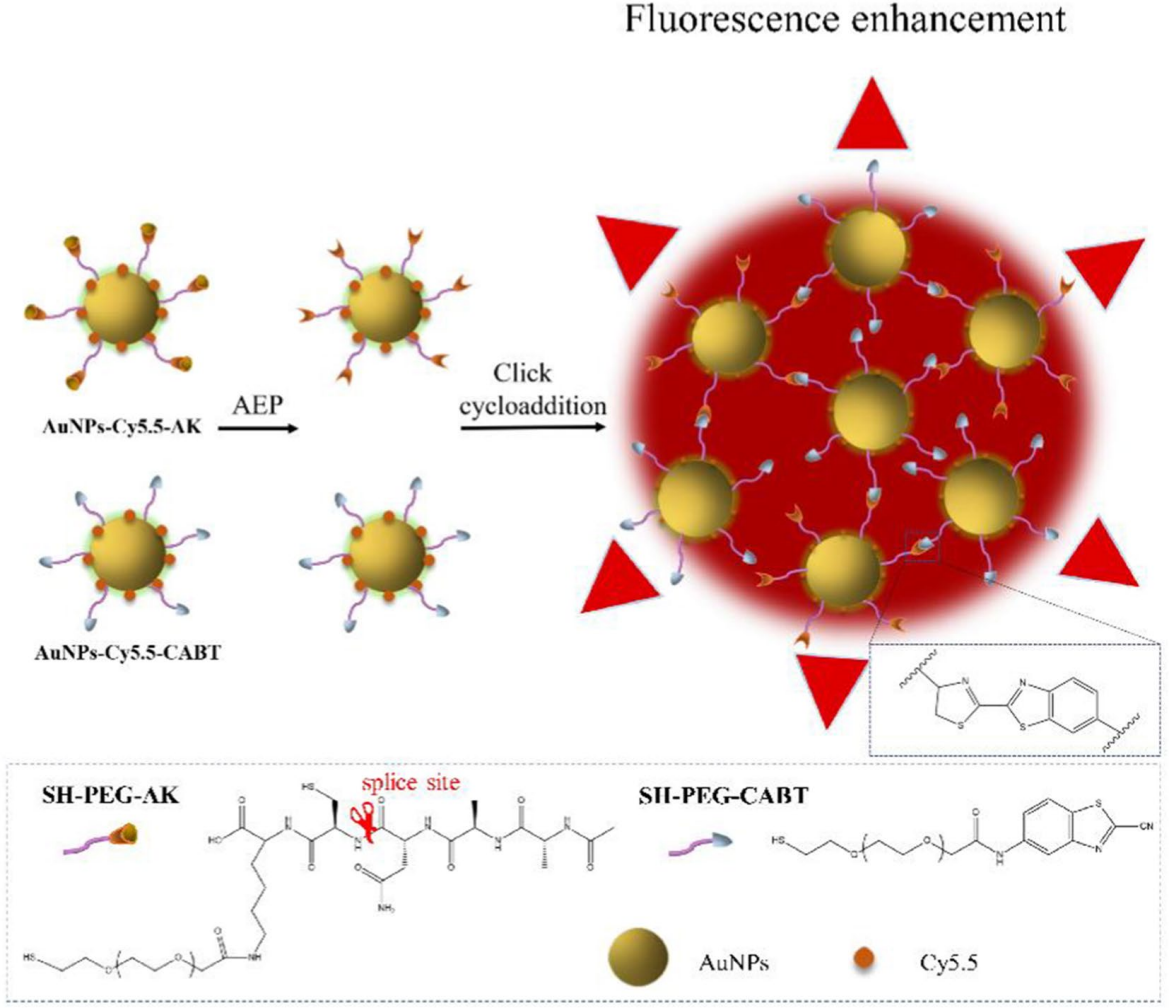
The mechanism of aggregation-based fluorescent enhancement. The AuNPs-Cy5.5-AK contained Ala–Ala–Asn–Cys–Lys, which was cut by AEP to expose 1, 2-thiolamino group of cysteine. The click cycloaddition occurred between the 1, 2-thiolamino group of AuNPs-Cy5.5-AK and cyano group of AuNPs-Cy5.5-CABT, leading to aggregation of AuNPs and strong fluorescent emission. Inset showing structures of SH-PEG-CABT and SH-PEG-AK and the splice site. This scheme was adapted with permission from (ACS Nano 2016, 10, 11, 10086–10098) [26]. Copyright © 2016 American Chemical Society)

In order to use an imaging method to measure brain AEP activity in live animals, we here employed gold nanoparticles (AuNPs) as carriers and designed an aggregation-based fluorescent enhancement approach, in which the modified AuNPs can be selectively triggered by AEP to aggregate and emit strong fluorescence. As illustrated in Scheme 1, the Cy5.5 conjugated AuNPs were modified with Ala–Ala–Asn–Cys–Lys, which could expose its 1, 2-thiolamino group on cysteine due to AEP-catalyzed hydrolysis. The AuNPs-Cy5.5-CABT contained 2-cyano-6-aminobenzothiazole (CABT). The cyano groups of AuNPs-Cy5.5-CABT could readily react with the 1, 2-thiolamino groups via click cycloaddition [26], leading to aggregation of AuNPs-Cy5.5-A&C and fluorescence enhancement. We characterized progression of AEP-related pathology in an AD transgenic mice model APPswe/PS1dE9 (APP/PS1) [27], then applied AuNPs-Cy5.5-A&C, which contained AuNPs-Cy5.5-AK and AuNPs-Cy5.5-CABT, to these mice and conducted live brain imaging. The aim of this study is to determine whether in vivo imaging of brain AEP can predict AD pathology.

## Materials and methods

### Reagents and materials

Chloroauric acid (HAuCl4) was purchased from Sinopharm Chemical Reagent Co., Ltd (Shanghai, China). Carboxylic acid PEG Thiol (SH-PEG-COOH) and SH-PEG-Cy5.5 were from Hunan Huateng Pharmaceutical Co., Ltd (Changsha City, China).The AK peptide (Ala–Ala–Asn–Cys–Lys) was customized by PHTD Peptide Co., Ltd (Zhengzhou, China). 2-Cyano-6-aminobenzothiazole (CABT) was customized by Shanghai Chemical Pharm-Intermediate Tech. Co., Ltd (Shanghai, China). Other chemical reagents were from Aladdin Bio-chem Technology Co., Ltd (Chengdu, China). The AEP inhibitor 7-Morpholin-4-yl-benzo[1,2,5]oxadiazol-4-ylamine (δ-secretase inhibitor 11, PubChem CID: 1095027) was purchased from J&K Scientific Ltd. Beijing, China. It was dissolved in dimethyl sulfoxide (DMSO) to make a stock solution and then diluted in a 0.9% NaCl solution containing gum Arabic for systemic treatment. The recombinant AEP and primary antibody for mature AEP were all from R&D Systems Inc (MN, USA). Amyloid β-protein fragment 25–35 (Aβ_25–35_) and Atorvastatin were from Sigma-Aldrich (St. Louis, MO, USA). The LysoTracker Green DND-26 for staining lysosomes in live cells was purchased from Thermo Fisher Scientific (Carlsbad, CA, US).

### Synthesis of SH-PEG-AK and SH-PEG-CABT

The AEP-responsive functional fragments, SH-PEG-AK and SH-PEG-CABT, were synthesized as previously reported [26]. Briefly, 52.1 mg SH-PEG-COOH (0.01 mmol) was dissolved in 2 mL DMF and then activated by EDC (4.11 mg) and NHS (2.59 mg) for 2 h at room temperature. Subsequently, 5 mg alanine–alanine–asparagine–cysteine–lysine (AK) was introduced to reaction system on ice surface. The system was further maintained for another 8 h at room temperature in dark. 99.2 mg SH-PEG-COOH was dissolved in 2 mL DMF and then activated with EDC (7.91 mg) and NHS (4.72 mg). After activation for 2 h at room temperature, 3.72 mg 2-Cyano-6-aminobenzothiazole (CABT) in DMF (5 mL) was introduced to the reaction system. The system was further reacted at room temperature for 8 h in dark. Finally, SH-PEG-AK and SH-PEG-CABT were purified by dialysis method using a bag filter (MW = 5000) and further lyophilized under vacuum.

### Preparation of AuNPs-Cy5.5-AK or AuNPs-Cy5.5-CABT

AuNPs were synthesized as previously described [28]. 1.5 mL HAuCl4 solution (20 mg mL^−1^) was diluted in 300 mL deionized water and heated to boiling under vigorous stirring. Then 12 mL sodium citrate solution (1%) was added into the boiling solution. The mixed solution was kept boiling until its color turned to wine red and the concentration of AuNPs was approximately 48 μg mL^−1^. To obtain AuNPs-AK or AuNPs-CABT, 20 mL AuNPs was incubated with 100 µL SH-PEG-AK (1 mg mL^−1^) or SH-PEG-CABT (1 mg mL^−1^) at 37 °C for 8 h. To obtain probes AuNPs-Cy5.5-AK and AuNPs-Cy5.5-CABT, the fluorescent tag Cy5.5 were conjugated to AuNPs (AuNPs-Cy5.5) as previously described [26]; subsequently, AuNPs-Cy5.5 were incubated with SH-PEG-AK or SH-PEG-CABT using the same procedure. Fluorescent intensity of Cy5.5-tagged AuNPs-AK or AuNPs-CABT was measured by a SpectraMax® M2e multi-mode microplate reader (San Jose, CA, USA).

### AEP triggered aggregation of AuNPs-A&C

Blood of the C57BL/6 mice was centrifuged at 500×*g* for 5 min at 4 °C to collect the plasma. AuNPs-AK (1 mL) and AuNPs-CABT (1 mL) were centrifugalized together at 16,000×*g* for 10 min at 4 °C. After removing the supernatant, the AuNPs-AK and AuNPs-CABT were re-suspended together in 1 mL HEPES containing 20% mouse plasma for 24 h. The concentration of AuNPs was 46.36 µg mL^−1^. Then AuNPs-A&C (1 mL) were further incubated at 37 °C, with or without adding 2.5 μL of AEP (1 mg mL^−1^) under different pH conditions. The hydrodynamic diameter of AuNPs-A&C was monitored using dynamic light scanning (DLS). Furthermore, transmission electron microscopy (TEM) images of AuNPs-A&C were observed using a JEM-1400 (Jeol Ltd., Tokyo, Japan). Atorvastatin (Ato) at 20 μM was used to inhibit AEP activity.

### Animals and ethical statement

Animal studies are reported in compliance with the ARRIVE guidelines [29]. Male APPswe/PS1dE9 transgenic mice (referred to as APP/PS1) and their wild-type (WT) littermates were purchased from Model Animal Research Center of Nanjing University, Nanjing, China. Male C57BL/6 mice were from Shanghai SLAC Laboratory Animal Co., Ltd (Shanghai, China). Animals were housed in the pathogen-free animal facility of the laboratory animal department at Shanghai Jiao Tong University School of Medicine (SJTU-SM). Animals were under a 12 h/12 h light/dark cycle and at 24 ± 2 °C, with free access to water and a standard rodent diet. Animal experimental procedures was approved by the Animal Experimentation Ethics Committee and Institutional Animal Care and Use Committee (IACUC) at SJTU-SM, and carried out strictly in accordance with the guideline of Association for Assessment and Accreditation of Laboratory Animal Care (AAALAC).

For treatment experiment, 5-month age APP/PS1 mice were randomly divided into two groups (n = 15/group): δ-secretase inhibitor 11 treatment and vehicle treatment. The δ-secretase inhibitor 11 was given to mice once daily via oral gavage (10 mg kg^−1^) over a period of 3 months. To collect brain tissue, mice were deeply anesthetized with 4% isoflurane (RWD Life Science, Shenzhen, China) and then decapitated.

### Cell culture and treatment

Human glioblastoma cell lines A172, U251 and rat glioma C6 cells were from American Type Culture Collection (ATCC) (Rockville, Maryland, USA). The pediatric glioblastoma cell line SF188 was kindly provided by Dr. Yu-Jie Tang (Shanghai Jiao Tong University School of Medicine, China) and Dr. Stefan Pfister (DKFZ, Germany) [30]. Cells were cultured in Dulbecco’s Modification of Eagle’s Medium (DMEM) (Gibco, Carlsbad, CA, USA) containing l-glutamine and 10% fetal bovine serum (FBS) (Gibco). They were plated in 6-well cell culture plates with 2.5 × 10^5^ cells per well and used at less than 30 passages. To check cellular uptake of probes, AuNPs-Cy5.5-AK, AuNPs-Cy5.5-CABT and AuNPs-Cy5.5-AC were dissolved in culture medium and the equivalent administration dose of Cy5.5 was 2 μg mL^−1^. After 24-h incubation, cells were subjected to flow cytometry analysis or immunofluorescent staining. Cells were imaged using a Leica SP8 confocal microscope (Leica Microsystems, Germany). Flow cytometry analysis of intracellular fluorescence was performed using the Coulter CytoFlexS flow cytometer (Beckman Coulter, CA, USA). Cell viability was assessed by using a Cell Counting Kit-8 (DOJINDO, Japan). To label lysosomes, C6 cells were loaded with 100 nM LysoTracker Green DND-26 (green) for 30 min and imaged thereafter.

Primary cortical neurons were from E15 to E17 female mice. Embryos were harvested acutely from pregnant C57BL/6 mice sacrificed by isoflurane anesthesia and cervical dislocation. Cortical neurons were plated on 35 mm poly-d-lysine and laminin coated dishes (Corning, NY, USA) at the density of 3 × 10^5^cells per ml in Neurobasal media (Invitrogen, CA, USA) supplemented with 2% B-27 (Invitrogen) and l-glutamine (0.5 mM). After 7 days in vitro (DIV), half of the medium was changed and the β-Amyloid treatment was performed 11 to 14 DIV. Aβ_25–35_ was dissolved in ultrapure water at a concentration of 50 mM as a stocking solution, and then divided into aliquots and stored at − 20 °C. Before use, Aβ_25–35_ was incubated at 37 °C for 7 days to obtain aggregated diffusible oligomers, and then diluted in the medium to the indicated concentration. Thereafter, neurons were incubated with AuNPs-Cy5.5-AK, AuNPs-Cy5.5-CABT or AuNPs-Cy5.5-AC (the equivalent administration dose of Cy5.5 at 2 μg mL^−1^) for 24 h then imaged with a Leica SP8 confocal microscope.

### Enzymatic activity assay

Recombinant mouse AEP (R&D Systems, Inc. MN, USA) was diluted to 50 μg mL^−1^ in activation Buffer (0.1 M NaOAc, 0.1 M NaCl, pH 4.5) and incubated for 6 h at 37 °C with or without AEP inhibitor. Then diluted to 2 ng μL^−1^ in assay buffer (50 mM MES, 250 mM NaCl, pH 5.5). Loaded 50 μL of 2 ng μL^−1^ AEP in the plate, and started the reaction by adding 50 μL of 200 μM Substrate: Z-Ala-Ala-Asn-AMC (Bachem AG, Switzerland). Substrate was also diluted with assay buffer. Included a Substrate Blank containing Assay Buffer and Substrate. Read at excitation and emission wavelengths of 380 nm and 460 nm (top read), respectively, in kinetic mode for 45 min. Tissue homogenates (10 μg) were incubated in 200 μL assay buffer containing 20 μM AEP Substrate and assayed as above description. The activity of AEP was expressed as the reading at 45 min minus the first reading.

### ELISA

To measure Aβ concentration, the mouse brain tissue were homogenized in buffer (5 M guanidine HCl diluted in 50 mM Tris–HCl, pH 8.0) and incubated at room temperature for 3 h. Then the samples were diluted with cold reaction buffer (phosphate buffered saline with 5% BSA and 0.03% Tween 20, supplemented with protease inhibitor cocktail) and centrifuged at 16,000×*g* for 20 min at 4 °C. The supernatant were assayed by human Aβ_1–40_ and Aβ_1–42_ ELISA kits (^#^KHB3481 and ^#^KHB3544, Invitrogen) according to the manufacturer’s instructions.

### Western blot analysis

Brain tissue were collected and lysed in RIPA buffer (Beyotime, Nanjing, China) supplemented with protease inhibitor (Beyotime). Equal protein extracts (30 μg protein per lane) were separated by SDS-PAGE and electrophoretically transferred to polyvinylidene difluoride membranes (Millipore, Bedford, MA, USA). Then, the membranes were incubated with anti-AEP antibodies (R&D Systems, Inc. Minneapolis, MN, USA) at 4 °C overnight, followed by incubation with *IRDye 680LT* fluorescent *secondary* antibody (LI-COR Biosciences, Lincoln, NE, USA). Proteins were visualized using the Odyssey Fc Imaging System (LI-COR Biosciences, Lincoln, NE, USA). Mouse β-action antibody was used as protein loading controls.

### Intracerebroventricular injection and controlled cortical impact (CCI) injury

Mice were anaesthetized with 3% isoflurane (RWD Life Science, Shenzhen, China) in a mixture of 30% O_2_ and 70% N_2_O. After induction of anesthesia, 1.5% isoflurane was maintained, and body temperature was kept at 37 ± 0.5 °C by a heating pad. Mouse head was restricted with a stereotactic injection instrument (RWD Life Science), allowing a precise coordinate setting. After shaving the hair and exposing the skull, one small hole (0.3 mm posterior to bregma, 1.0 mm left to the midline) was drilled. A total of 3 µL gold nanoparticles (45 µg mL^−1^ in deionized water) were infused into lateral ventricle (3 mm ventral to the dura) by using a Hamilton 80330 701 μL needle syringe (Hamilton Company, Reno, NV). The injection speed is 0.5 µL min^−1^. The needle was pull out after a 15-min waiting period.

The experimental operation of CCI in mice was performed as previously described with minor modification [31]. After a midline skin incision, a circular craniotomy (3.5-mm diameter) was performed over the right parietal cortex, between lambda and bregma, 2.0 mm lateral right to the midline. To induce CCI to the exposed cortex, we used the PCI3000 precision cortical impactor (Hatteras Instruments, Cary, NC) to drive a 2.0-mm diameter flat-tip. The position of the tip was held within the center of craniotomy, and angled to be vertical to the dura surface. The tip impacted dura surface with a velocity = 3 m s^−1^, contact time = 150 ms, and depth = 1.0 mm. After injury surgery, the skin incision was sutured, anesthesia was discontinued, and mice were removed from the stereotaxic frame and maintained in a humidity-controlled incubator (Lyon Technologies, California). Sham-operated control mice only received procedure of skin incision and circular craniotomy.

### Morris water maze test

Mice were trained in a round water pool with extra‐maze cues. Each animal received four training trials per day for 5 consecutive days, to learn to find the hidden platform located 1.5 cm below the water surface. In each trial, mice were given 60 s to find the invisible platform in one of four different positions. The escape latency (the time required to find and climb onto the platform) was recorded for up to 60 s. After each trial, mice were dried and kept in a warm cage. The probe test was conducted 24 h after the last training. Water maze test data were analyzed by an investigator who was blinded to the treatment.

### Immunohistochemical staining of Aβ plaque

Mice were deeply anesthetized and perfused with 4% paraformaldehyde (PFA) in phosphate-buffered saline (PBS) through the heart. The whole brain was removed, embedded in paraffin, and sectioned at 4 μm. For immunohistochemistry, antibody 6E10 (Covance, USA) was used to stain Aβ deposition and the standard ABC-DAB method was performed. Sections were finally counterstained with hematoxylin, and images were taken and analyzed using Leica Qwin software. Quantification was carried out on six slices of each brain spaced 120 μm apart to estimate the average intensity of the immunostaining per unit area. Quantification and analysis was conducted by a person who was blinded to the treatment.

### Live animal brain imaging

Brain AEP activity in live animals were imaged by using probe AuNPs-Cy5.5-A&C under an IVIS Spectrum Imaging System (Perkin Elmer, Waltham, MA, USA), which captured the fluorescence signal (ex: 685 nm, em: 710 nm) emitted from AuNPs-Cy5.5-A&C. Prior to imaging, mice were anesthetized with inhalation of isoflurane gas (RWD Life Science); the isoflurane was balanced with oxygen, dialed to 2.0% for the induction of anesthesia and 1.0% for maintenance. Mice with traumatic brain injury (TBI) were injected with AuNPs-Cy5.5-A&C (1.5 mg kg^−1^) aqueous solution via intravenous tail vein. As for APP/PS1 mice, AuNPs-Cy5.5-A&C (3 µL) was directly injected into the left lateral ventricle of the brain. Images were and captured at 0.5, 1, 2, 4 and 8 h following injection. Signal intensity was quantified within a region of interested over the head, as defined by Living Image software. The data were analyzed using Living Image 4.4.5 software (Perkin Elmer, RRID: SCR_014247).

### Statistical analysis

Data were expressed as mean ± SEM and analyzed by Prism 7 software (La Jolla, CA). The concentration of the inhibitor yielding half-maximal inhibition (IC_50_) of AEP activity was calculated using the equation:

$${\text{Fractional Enzymatic Activity}}\,\left( {\% \;{\text{of control}}} \right) = {\text{Bottom}} \,+\, ({\text{Top}}\,-\,{\text{Bottom}})/(1 \,+\, 10^{{(({\text{LogIC}}50 - C)*n)}} ),$$

where *C* is the logarithm of inhibitor concentration and *n* is the Hill coefficient. The statistical difference between two independent groups was analyzed by unpaired Student’s *t*-test. And the data of more than two groups was assessed by the parametric one-way ANOVA followed by a Tukey’s post-hoc test. Post hoc tests were conducted when the F value achieved the necessary level (*P* < 0.05) and there was no significant variance inhomogeneity. For data of Morris water maze test, a two-way ANOVA repeated measures was used to compare acquisition data of the two groups. A Student’s *t*-test was used to compare time spent and distance travelled in target quadrant between two groups. Differences were considered to be significant when *P* < 0.05.

## Results

### Characterization of AEP-responsive gold nanoparticles

**Fig. 1:**
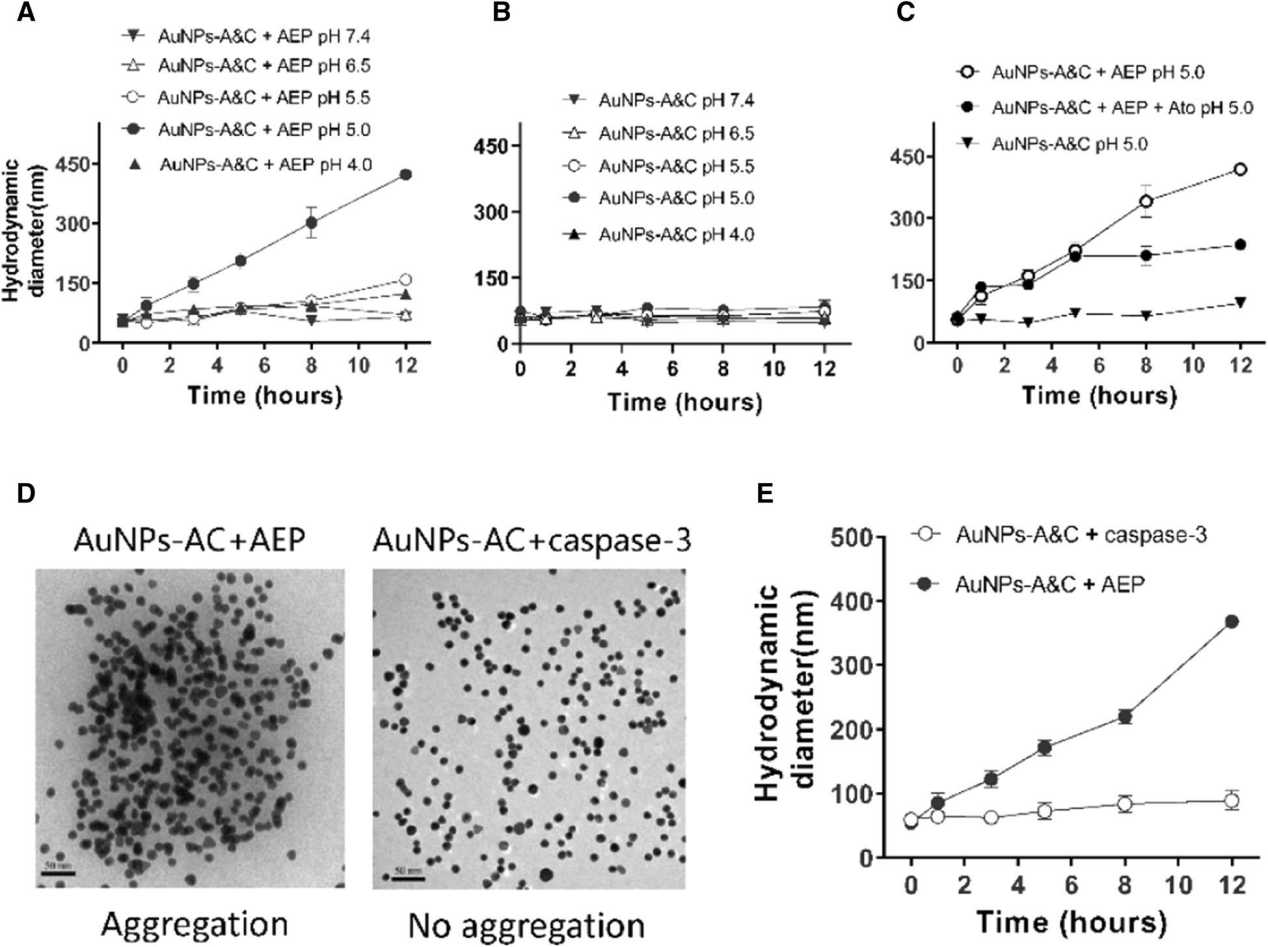
AEP-triggered size increase of gold nanoparticles (AuNPs). **A** Hydrodynamic diameter of AuNPs-A&C elicited by AEP (1 mg mL^−1^) in HEPES buffer with various pH values, for 12 h. **B** Diameter of AuNPs-A&C under various pH in the absence of AEP. **C** In pH 5.0 HEPES buffer, hydrodynamic diameter of AuNPs-A&C incubated with AEP (1 mg mL^−1^) for 12 h. 20 μM Atorvastatin (Ato) was used to inhibit AEP. The size of AuNPs-A&C in absence of AEP was also examined. **D** TEM images of AuNPs-A&C particles incubated with AEP (1 mg mL^−1^) or cleaved-caspase 3 (1 mg mL^−1^) for 12 h. **E** Hydrodynamic diameter of AuNPs-A&C incubated with AEP or cleaved-caspase 3 for 12 h. For above data, n = 3 independent experiments

We modified AuNPs with an AEP sensitive peptide Ala–Ala–Asn–Cys–Lys (AK), which can be cleaved by AEP to expose 1, 2-thiolamino group. In the presence of AEP, the click cycloaddition occurred between the 1, 2-thiolamino group and the cyano group of AuNPs-CABT, resulting in aggregation of AuNPs-AK and AuNPs-CABT (called AuNPs-A&C). DLS analysis showed that the particle size of AuNPs was around 30 nm; size of AuNPs-AK or AuNPs-CABT was 40–50 nm (Table 1). To test the responsiveness of AuNPs-A&C to AEP, we mixed AuNPs-AK and AuNPs-CABT together and pre-incubated them in HEPES buffer containing 20% mouse plasma for 24 h. Then, AEP was added to trigger the click cycloaddition between AK and CABT and induce aggregation of AuNPs-A&C. Because activation of AEP needs an acidic condition [17], we incubated AuNPs-A&C with AEP in at pH values of 7.4, 6.5, 5.5, 5.0, and 4.0 to discriminate the efficiency of AEP-triggered reaction. The initial size of AuNPs-A&C was 50–60 nm and increased to 422.2 ± 9.69 nm after a 12-h incubation with AEP at pH 5.0; but didn’t gain so dramatic aggregation under other pH conditions (Fig. 1A). Therefore, pH 5.0 is the best condition for AEP to trigger aggregation of AuNPs-A&C. If AEP was absent, the size increase of AuNPs-A&C was completely abrogated no matter of the pH conditions (Fig. 1B).

**Table 1:** Physiochemical characterization of the formulations

Formulation	Particle size (nm)	PDI	Zeta potential(mV)
AuNPs	28.96 ± 0.22	0.180 ± 0.002	− 32.63 ± 1.397
AuNPs-AK	54.55 ± 1.14	0.224 ± 0.01	− 22.6 ± 0.31
AuNPs-CABT	44.37 ± 1.75	0.246 ± 0.003	− 12.63 ± 0.75

n = 3 independent experiments

*PDI* Polydispersity Index

To further confirm the responsiveness of AuNPs-A&C to AEP, we added an AEP inhibitor atorvastatin (Ato) to the incubation buffer and found that the size increase of AuNPs-A&C was greatly suppressed (Fig. 1C). These data proved that the click cycloaddition didn’t occur in the absence of AEP. Considering AEP is a kind of cysteine protease, we incubated another cysteine protease caspase-3 with AuNPs-A&C for 12 h. Under the transmission electron microscopy (TEM), AEP triggered a remarkable aggregation of AuNPs-A&C; but cleaved caspase-3 didn’t induce aggregation (Fig. 1D, E). So, AuNPs-A&C responded selectively to the protease activity of AEP.

### The responsiveness of AuNPs-Cy5.5-A&C to AEP

We conjugated the fluorescent tag Cy5.5 to AuNPs-AK and AuNPs-CABT respectively to get AuNPs-Cy5.5-AK and AuNPs-Cy5.5-CABT. The activity based-AEP probe AuNPs-Cy5.5-A&C was obtained by mixing AuNPs-Cy5.5-AK and AuNPs-Cy5.5-CABT together. To test the responsiveness of AuNPs-Cy5.5-A&C to AEP, we incubated this probe in pH 5.0 HEPES buffer without or with AEP (1 mg mL^−1^) for 12 h (Additional file 1: Figure S1). AuNPs-Cy5.5-AK or AuNPs-Cy5.5-CABT alone was also incubated in the same buffer. Over the 12-h incubation, the fluorescence of these probes underwent a slow increase probably due to evaporation of the buffer and probe concentrating. Noticeably, the fluorescent intensity of AuNPs-Cy5.5-A&C was significantly augmented after incubation with AEP for 7 h, as compared with other groups (*P* < 0.01, one-way ANOVA). This enhancement was abrogated by an AEP inhibitor Atorvastatin (Ato, 20 μM) or in the absence of AEP. So, AuNPs-Cy5.5-A&C was able to emit strong fluorescence under the action of active AEP.

**Fig. 2:**
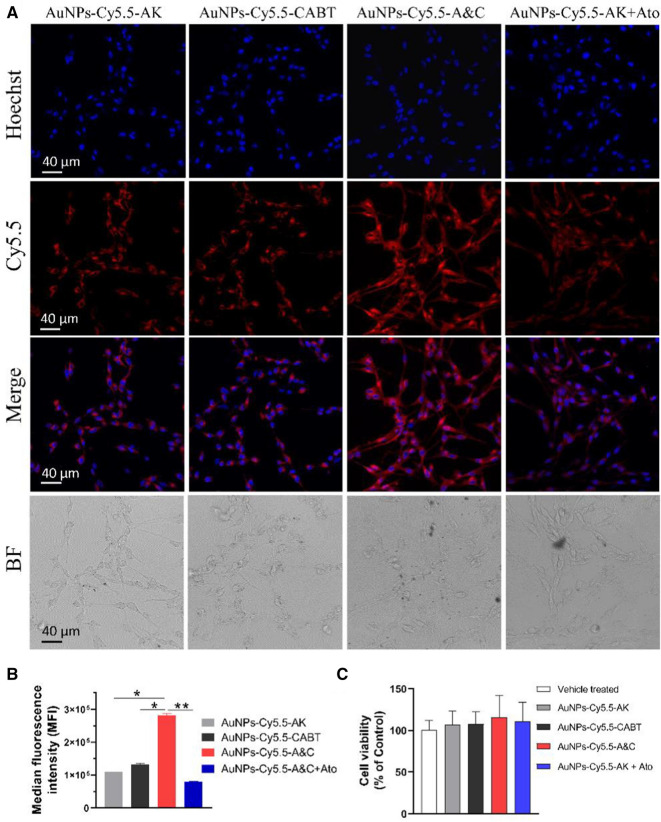
Fluorescence intensity of AuNPs-Cy5.5-A&C in C6 cells. **A** Image of C6 cells incubated with AuNPs-Cy5.5-A&C and other probes for 24 h. 20 μM Atorvastatin (Ato) was added to inhibit AEP activity. The bottom pictures were bright-field (BF) images of C6 cells of each group. **B** Fluorescent intensity of AuNPs-Cy5.5-A&C and other probes in C6 cells was measured by flow cytometry analysis. *P < 0.05, **P < 0.01 between groups as indicated; one-way ANOVA, n = 3 independent experiments. **C** Viability of C6 cells after 24-h incubation with various nanoparticles. n = 3 independent experiments

To determine whether AuNPs-Cy5.5-A&C can enter a cell and react with cellular AEP, we incubated this probe with C6 glioma cells, which have considerable level of AEP expression [26]. The control probe AuNPs-Cy5.5-AK or AuNPs-Cy5.5-CABT alone was also incubated with C6 cells. Fluorescent intensity of these probes in C6 cells was measured and analyzed with flow cytometry. After a 24-h incubation, AuNPs-Cy5.5-A&C permeated into cytoplasm and emitted strong red fluorescence (Fig. 2A), suggesting that AuNPs-Cy5.5-A&C aggregated due to AEP triggered click cycloaddition between AK and CABT. In contrast, C6 cells incubated with either AuNPs-Cy5.5-AK or AuNPs-Cy5.5-CABT showed much weak fluorescence (Fig. 2A, B). If C6 cells were pre-treated with an AEP inhibitor Ato (20 μM), the fluorescence of AuNPs-Cy5.5-A&C was greatly suppressed. The cellular viability of C6 cells was not suppressed by 24-h incubation with these probes (Fig. 2C). So, AuNPs-Cy5.5-A&C was able to react with cellular AEP and emit strong fluorescence.

**Fig. 3:**
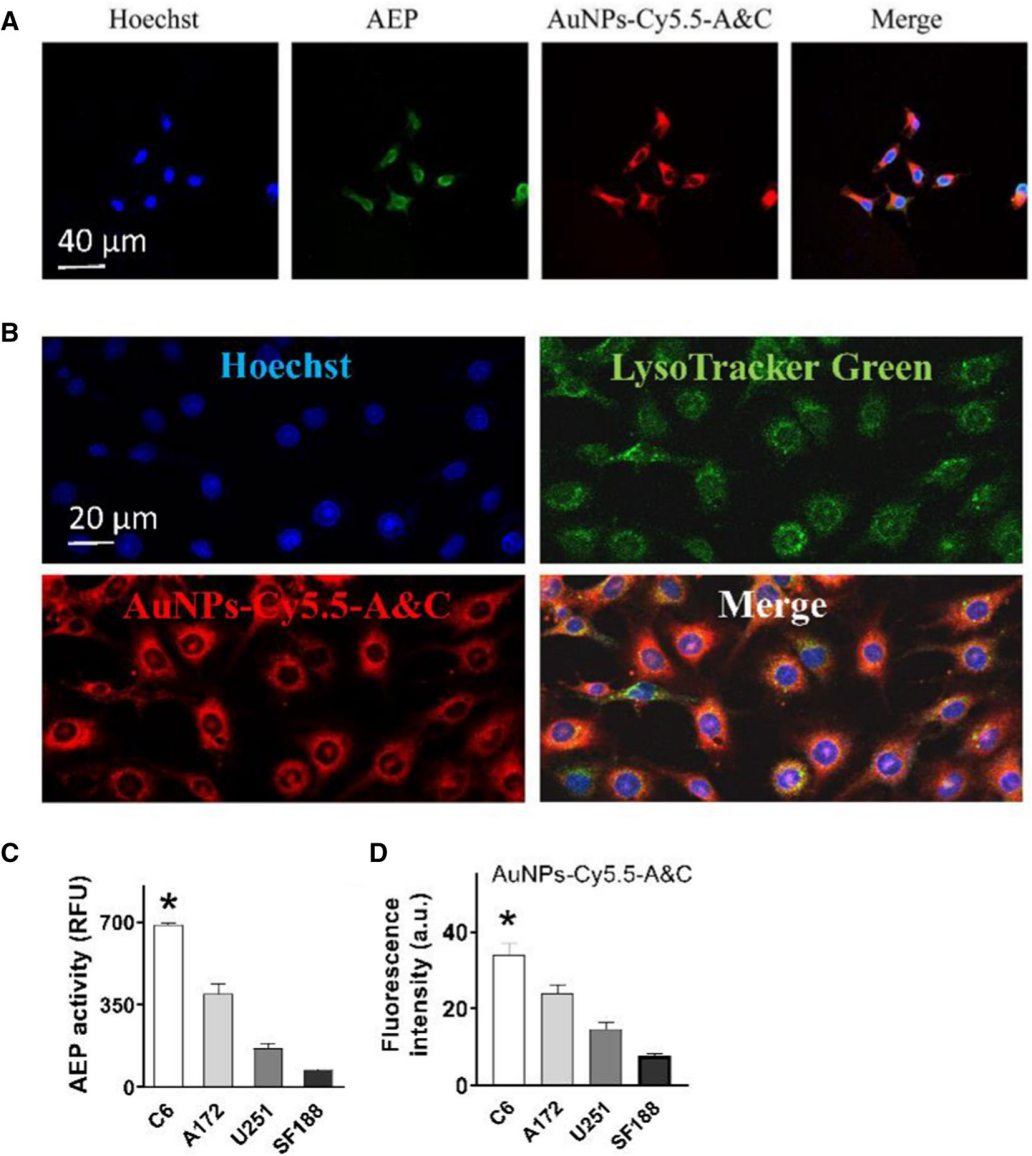
Cellular uptake of AuNPs-Cy5.5-A&C and its fluorescence intensity in different cell lines. **A** Uptake of AuNPs-Cy5.5-A&C into cytoplasm after incubation with C6 cells for 24 h. Immunofluorescence images of AEP was co-localized with AuNPs-Cy5.5-A&C in cytoplasm. **B** AuNPs-Cy5.5-A&C was co-localized with LysoTracker Green DND-26 in C6 cells. **C** Enzymatic activity of AEP in C6 cells is higher than other cell lines. *P < 0.05, one-way ANOVA. n = 3 independent experiments. **D** Fluorescent intensity of AuNPs-Cy5.5-A&C in C6 cells is significantly higher than other cell lines. *P < 0.05, one-way ANOVA. n = 3 independent experiments, and 60–70 cells were imaged per experiment

Immunofluorescent staining further revealed that AuNPs-Cy5.5-A&C (red) was co-localized with AEP (green) in the cytoplasm of C6 cells (Fig. 3A). Given that cellular AEP becomes activated in the acidic environment of lysosomes [17], AuNPs-Cy5.5-A&C must at least enter lysosomes to react with AEP. We then incubated C6 cells with AuNPs-Cy5.5-A&C for 8 h and employed a lysosome-targeted fluorescent dye LysoTracker Green DND-26 to stain lysosomes for 30 min. Images of cells demonstrated that AuNPs-Cy5.5-A&C was co-localized with LysoTracker Green DND-26 (Fig. 3B), indicating that the probe got into lysosomes.

The next step was to determine whether fluorescent intensity of AuNPs-Cy5.5-A&C correlated with AEP activity in different cell lines. Human glioblastoma cell lines A172, U251 and SF188 were included together with C6 cells in this test. We found the enzymatic activity of AEP in C6 cells was the highest one, in A172 cells ranked the second and in U251 cells was the third (Fig. 3C). We incubated AuNPs-Cy5.5-A&C with these cell lines for 24 h then performed live cell imaging. The fluorescence emission of AuNPs-Cy5.5-A&C in C6 cells was highest, in A172 cells ranked the second and in U251 cells was the third (Fig. 3D), indicating that this probe’s fluorescent intensity correlated well with cellular AEP activity.

### Use of AuNPs-Cy5.5-A&C to detect AEP activity in Aβ-treated neurons

**Fig. 4:**
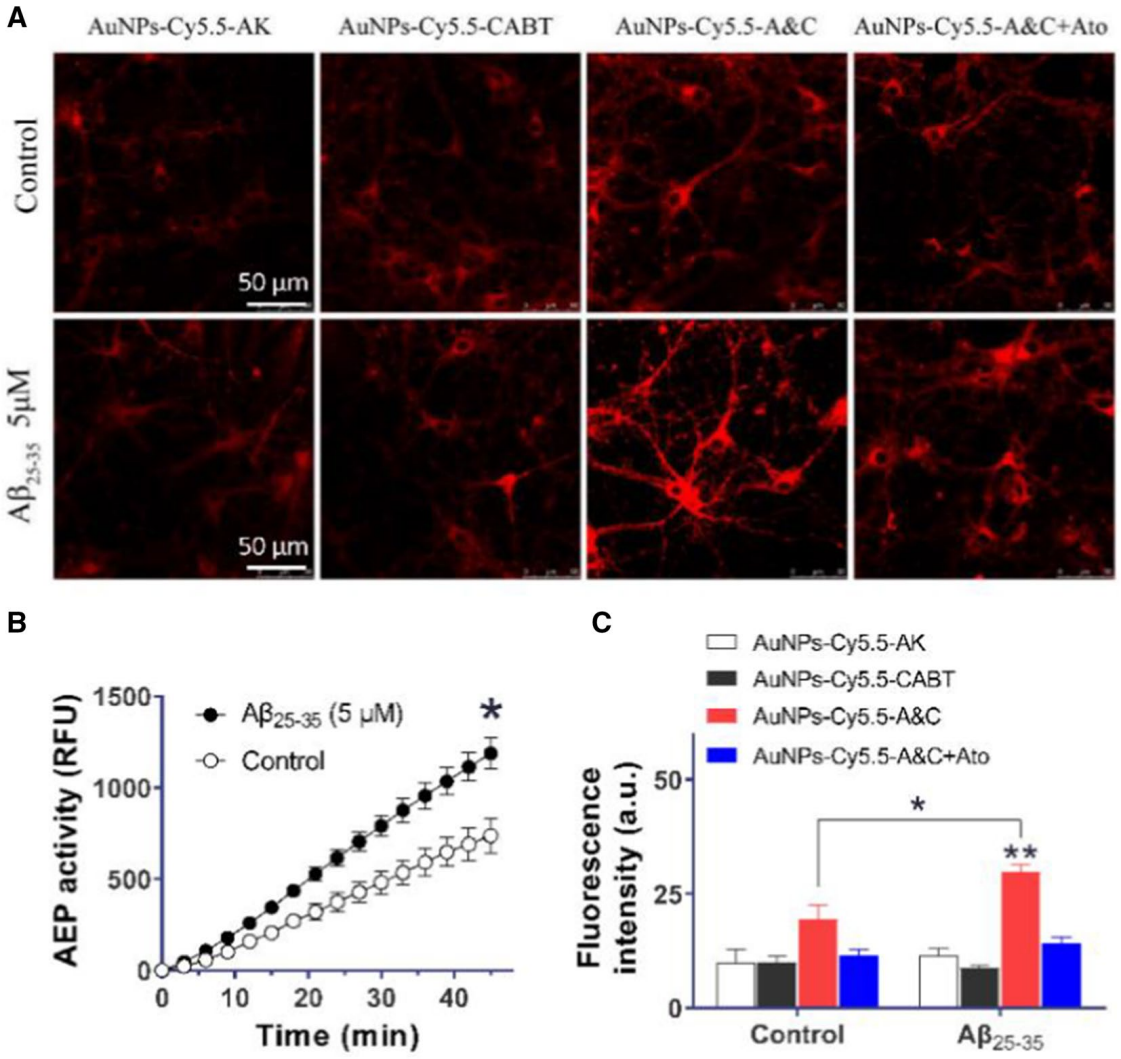
Fluorescence intensity of AuNPs-Cy5.5-A&C in cortical neurons. **A** Images of cortical neurons after incubation with AuNPs-Cy5.5-A&C or other probes for 24 h. Treatment with Aβ_25–35_ (5 μM) were for 24 h. Atorvastatin (Ato) at 20 μM was used to inhibit AEP. **B** The enzymatic activity of AEP in neurons. *P < 0.05, Aβ treatment versus control group. n = 3 independent experiments, Student’s *t*-test. **C** Fluorescent intensity of Cy5.5-tagged probes after a 24-h incubation with neurons. *P < 0.05, AuNPs-Cy5.5-A&C in Aβ-treated cells compared with control cells. Student’s *t*-test, n = 3 independent experiments. **P < 0.01, AuNPs-Cy5.5-A&C compared with other probes in Aβ-treated cells, one-way ANOVA. n = 3 independent experiments, 40–50 cells were imaged per test

Above results convinced us that the AEP probe AuNPs-Cy5.5-A&C can be applied to various cell lines for assessment of AEP activity. AEP has been reported to play a pivotal role in AD pathogenesis by cleaving APP and increasing Aβ generation; conversely, cytotoxic Aβ also enhances AEP activity [13]. We treated mouse cortical neurons with a soluble oligomer Aβ (5 μM) for 24 h and found that neuronal AEP activity was significantly elevated (Fig. 4B). Thereafter, we incubated these neurons with AuNPs-Cy5.5-AK, AuNPs-Cy5.5-CABT or AuNPs-Cy5.5-A&C for 24 h and performed live cell imaging (Fig. 4A). The fluorescence intensity of AuNPs-Cy5.5-A&C in Aβ-treated neurons was significantly augmented in comparison with control neurons (Fig. 4C). This enhanced fluorescence resulted from AEP activation because it was abrogated by the AEP inhibitor Ato (20 μM). The control probe AuNPs-Cy5.5-AK or AuNPs-Cy5.5-CABT alone didn’t show a fluorescence enhancement in Aβ-treated neurons. So, AuNPs-Cy5.5-A&C was able to monitor AEP activity in degenerative neurons.

### Use of AuNPs-Cy5.5-A&C to detect brain AEP activity in mice with TBI

**Fig. 5:**
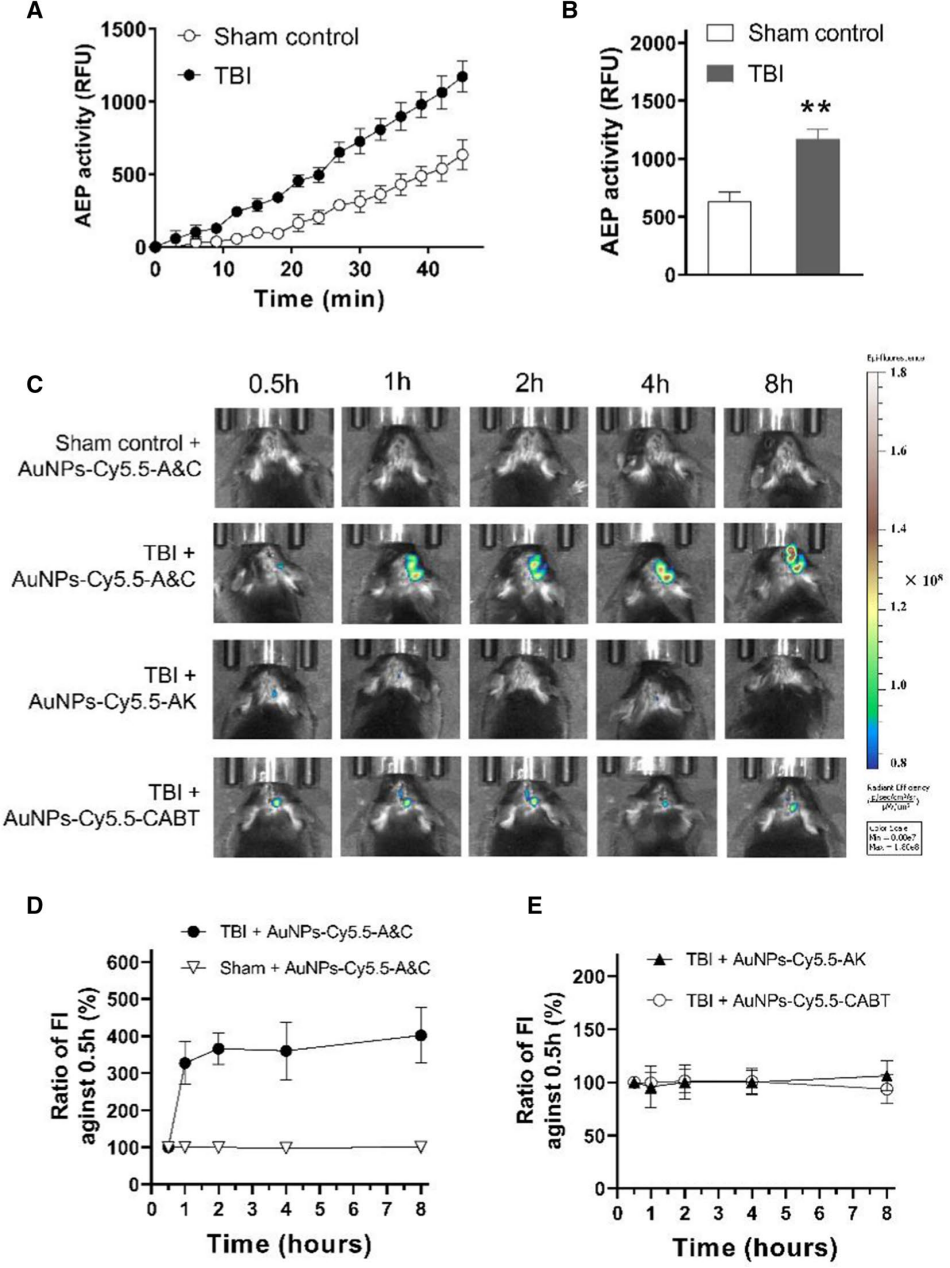
Brain AEP activity in TBI mice and in vivo brain imaging. **A**, **B** Activity of AEP in peri-contusional region measured 2 days after TBI. **P < 0.01 between two groups, Student’s *t*-test. **C** Live imaging of mouse heads at 0.5, 1, 2, 4, 8 h post intravenous injection of Cy5.5-tagged probes. **D**, **E** Ratio: the fluorescence intensity (FI) at each time point divided by FI at 0.5 h post injection of the probes. Above data, n = 6 mice per group

TBI activates AEP in brain tissue [15]. We found that 2 days after TBI, the activity of AEP was markedly enhanced in the peri-contusional region (Fig. 5A, B) of mouse brain. Thereupon we applied AuNPs-Cy5.5-A&C to these animals and examined whether it could monitor AEP activity in brains with TBI. AuNPs-Cy5.5-A&C were delivered into animal body via intravenous tail vein injection. The blood brain barrier (BBB) has been damaged by TBI [32], allowing the cell permeable probe AuNPs-Cy5.5-A&C to enter the brain. Using the IVIS Spectrum Imaging System, we imaged mouse brains with TBI and found the fluorescence intensity of AuNPs-Cy5.5-A&C was low at 0.5 h post intravenous injection, then markedly increased at 1, 2, 4 and 8 h (Fig. 5C, D). As for the sham-operated mouse brain, we didn’t observed fluorescence enhancement from 0.5 to 8 h after intravenous injection of AuNPs-Cy5.5-A&C. The control probes AuNPs-Cy5.5-AK or AuNPs-Cy5.5-CABT was also injected into TBI mice, but didn’t show fluorescence increase (Fig. 5E). No significant fluorescence emission was recorded from mouse body because the body hair was kept intact when mice were imaged (Additional file 1: Figure S2). The thick hair on the body occluded the fluorescence of probes. Above data indicated that live animal imaging with AuNPs-Cy5.5-A&C was able to detect elevated brain AEP as long as this probe got into brain tissue.

### Ageing-associated change of AEP in the brain of APP/PS1 mice

**Fig. 6:**
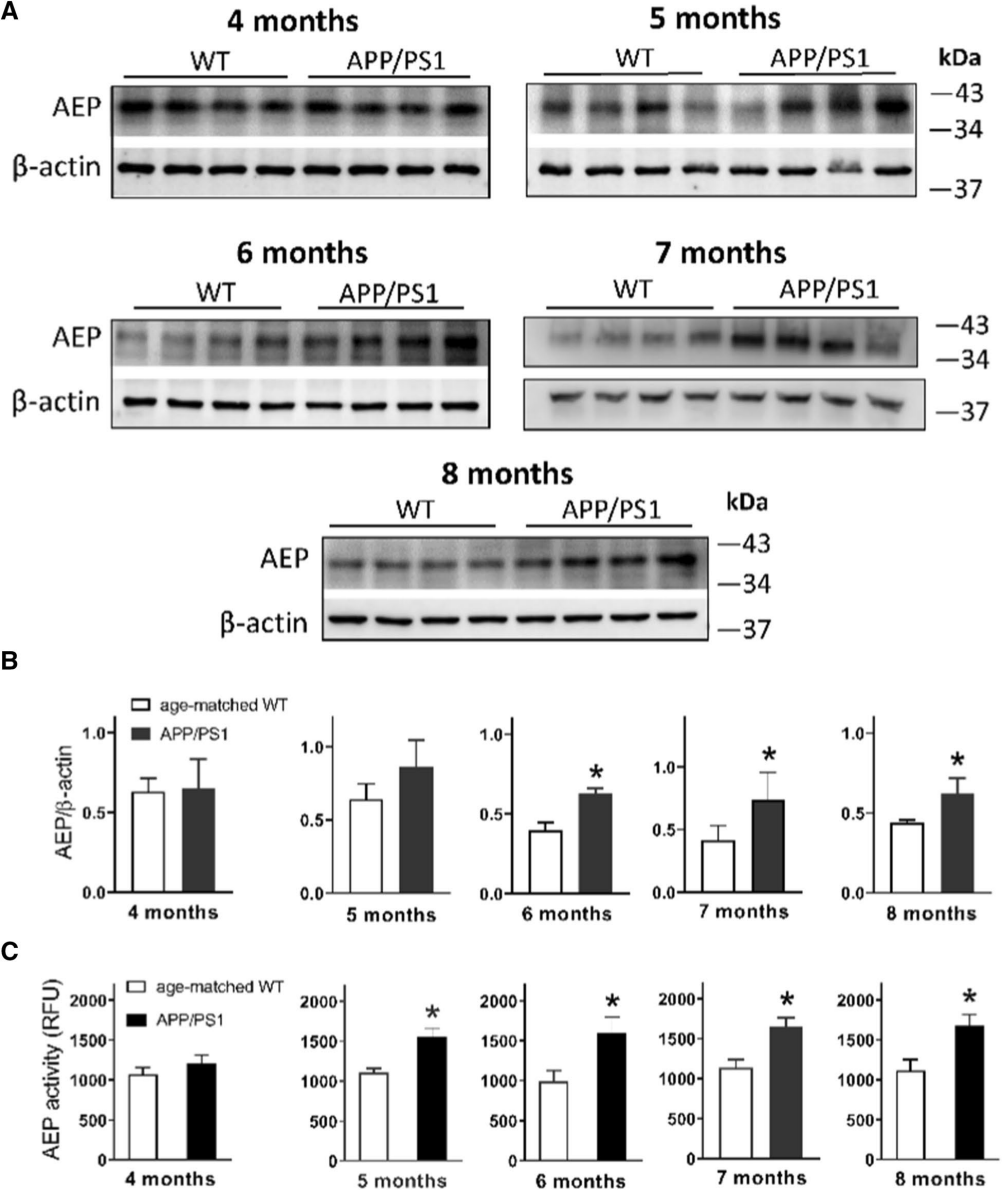
Protein expression and enzymatic activity of asparagine endopeptidase (AEP) in the brain. **A** AEP expression by western blot in brain tissue of APP/PS1 and WT mice with ages of 4 to 8 months. **B** Band gray density of AEP/β-actin and comparison between groups. *P < 0.05, student *t*-test, n = 4 per group. **C** Activity of AEP in brain tissue of APP/PS1 and WT mice with ages of 4 to 8 months. *P < 0.05, Student’s *t*-test, n = 5 per group

To determine ageing-associated change of AEP in the brain of APP/PS1 mice, we detected protein expression and activity of mature AEP in these AD model and age-matched control mice (4 to 8 months old). Whole brain tissue analysis revealed that the level of active AEP fragments in 4-month old APP/PS1 mice was similar to that in age-mated WT mice, and displayed a trend of increase at 5 months of age. The expression of AEP was significantly higher in APP/PS1 mice with 6–8 months of age than that in WT mice (Fig. 6A, B). Particularly, the enzymatic activity of AEP in APP/PS1 mice already reached a significantly higher level than WT mice at as early as at 5 months of age (Fig. 6C). This elevated AEP activity was also observed in 6 to 8-month old APP/PS1 mice.

### Cognitive function and Aβ deposition of APP/PS1 mice at different ages

To monitor the progressive cognitive decline in this AD model, Morris water maze test was performed on APP/PS1 mice with ages of 5, 6 and 8 months and age-mated WT mice. As for 5 and 6 months old mice, a two-way ANOVA analysis of latency to the escape platform generated a main effect of the training days (F (3.677, 44.12) = 66.04, *P* < 0.05) but no significant difference between groups (F (3, 12) = 0.6744, *P* > 0.05; Additional file 1: Figure S3A). The same analysis of swimming speed revealed main effect of training days (F (2.407, 28.89) = 29.04, *P* < 0.05) but not of groups (F (3, 12) = 0.6094, *P* > 0.05; Additional file 1: Figure S3B). The probe test was on day 6 after the acquisition period. The percentage of time spent and distance travelled in the target quadrant did not show significant difference between APP/PS1 and WT mice (Additional file 1: Figure S3C, D). These data imply that APP/PS1 mice of 5–6 months did not develop a deficiency in spatial memory. Until 8 months old, the water maze test revealed significant differences in latency over the training days (F (2.152, 12.91) = 4.614, *P* < 0.05) and an overall effect of group × training day (F (4, 24) = 2.887, *P* < 0.05; Additional file 1: Figure S3E). There was no difference in swimming speed between APP/PS1 and WT mice **(**Additional file 1: Figure S3F). However, 8-month old APP/PS1 mice travelled significantly less time and distance in the target quadrant than age-mated WT mice (Additional file 1: Figure S3G, H), indicating some impairment in spatial learning and memory.

The AD-like pathology in APP/PS1 mice mainly expresses Aβ toxicity [27]; we hereby detected Aβ plaque deposition by brain section staining with anti-Aβ antibodies 6E10. The immunoreactivity of 6E10 was almost absent in the hippocampus (Additional file 1: Figure S4A, B) and cortex (Additional file 1: Figure S4C, D) of 5-month-old APP/PS1 mice, began to appear at 6 months and increased to a significantly severe level at 8 months of age as compared with WT mice. The progressive development of Aβ plaque in AD mice was consistent with the timeline of memory loss.

### Pharmacological inhibition of AEP in APP/PS1 mice with δ-secretase inhibitor 11

Above data indicate that AEP activity in APP/PS1 mice brain began to increase at 5 months of age; this elevation precedes emergence of Aβ plaque and cognitive impairment. AEP played as a δ-secretase to cleave APP, facilitate β-secretase-mediated processing of APP fragments and increase Aβ production [12]. Inhibition of AEP by a δ-secretase inhibitor 11 (10 mg kg^−1^, p.o.) reduced Aβ production in 5xFAD mice [16]. We here asked whether AEP also played a mediating role in Aβ plaque formation of APP/PS1 mice. To answer this question, we gave δ-secretase inhibitor 11 to APP/PS1 mice to suppress brain AEP activity and intervene AD-like pathological progression.

**Fig. 7:**
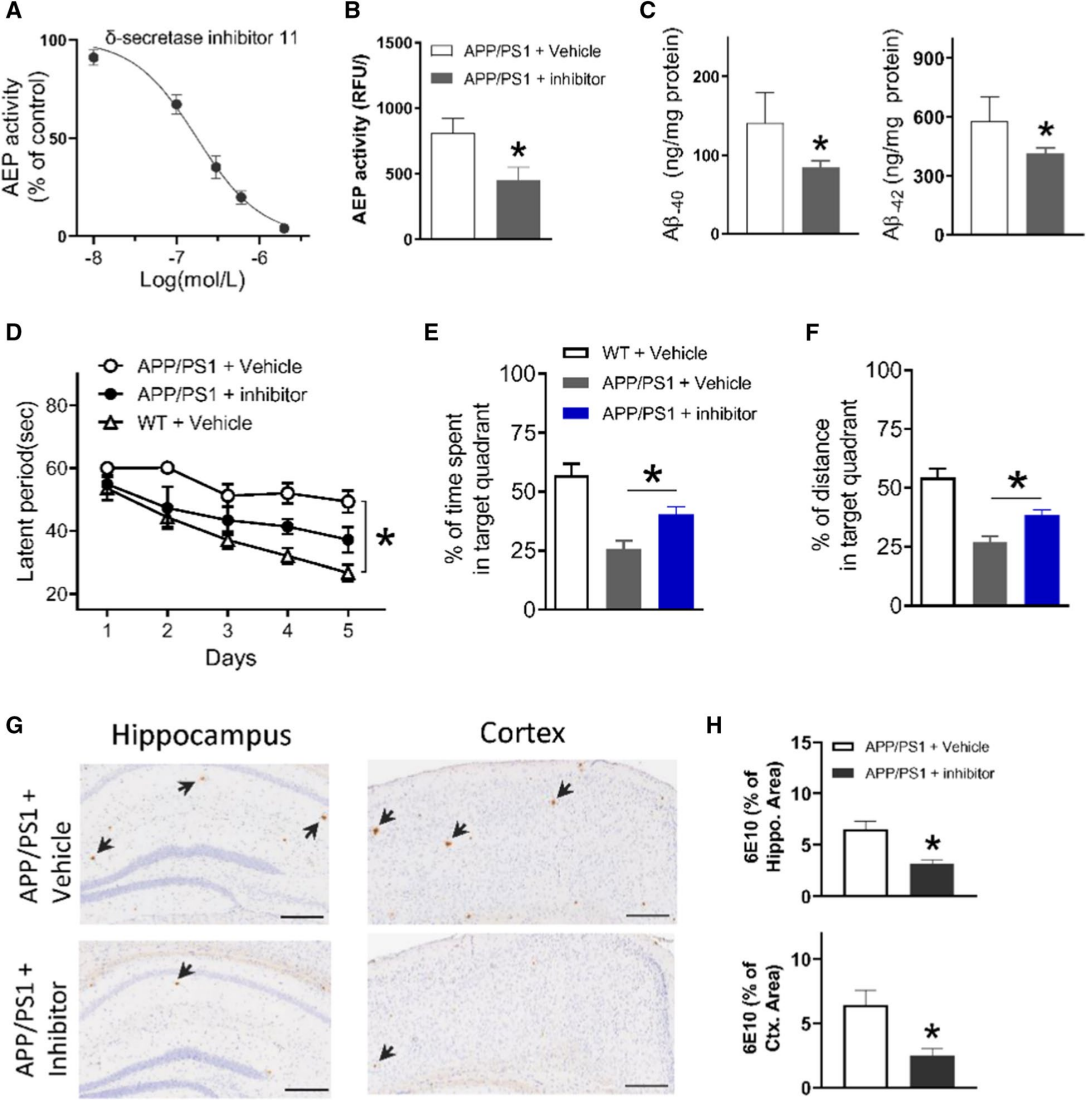
Therapeutic effect of δ-secretase inhibitor 11 on APP/PS1 mice. **A** Action of δ-secretase inhibitor 11 on AEP activity. **B**, **C** AEP activity, Aβ_1–40_ and Aβ_1–42_ in brain tissues of APP/PS1 mice treated with vehicle or δ-secretase inhibitor 11 (10 mg kg^−1^, p.o.) for 3 days. *P < 0.05, unpaired Student’s *t*-test; n = 5 mice per group. **D** The latency to the escape platform over the 5-day acquisition training. *P < 0.05, significant time and group effects, two-way ANOVA analysis. n = 10 mice per group. **E**, **F** The percentage of time and distance travelled in the target quadrant. *P < 0.05 between groups, one-way ANOVA, n = 10 mice per group. **G** 6E10 (arrowhead) in the hippocampus and cortex of APP/PS1 mice treated with δ-secretase inhibitor 11 or vehicle. Scale bar = 250 μm. **H** The average percentage of area occupied by 6E10-positive staining. *P < 0.05, Student’s *t*-test; n = 5 mice per group

This δ-secretase inhibitor 11 (previously named compound 11) was reported to inhibit AEP activity with an IC_50_ value of 0.70 ± 0.18 μM [16]. This effect is 46- to > 282-fold more potent than inhibition over other cysteine proteases, such as caspase-3, caspase-8, and cathepsin-S. This compound can cross murine blood–brain-barrier (BBB) after oral administration and did not incur long-term systemic toxicity [16]. Our in vitro assay showed that δ-secretase inhibitor 11 inhibited AEP activity with an IC_50_ value of 0.28 ± 0.03 μM (Fig. 7A). We treated 5-month old APP/PS1 mice with δ-secretase inhibitor 11 (10 mg kg^−1^, p.o.) or vehicle once daily for 3 days then collected brain tissue. We found that brain AEP activity was significantly decreased by δ-secretase inhibitor 11 (Fig. 7B). Moreover, the concentrations of Aβ_1–40_ and Aβ_1–42_ in brain lysates significantly decreased due to AEP inhibition (Fig. 7C). Thereafter, we continued to treat remaining animals for 3 months and performed water maze test on them. The two-way ANOVA analysis of latency periods revealed significant effects of training days (F (2.858, 60.01) = 16.50, P < 0.05) and groups (F (2, 21) = 15.27, P < 0.05; Fig. 7D). In the probe test, δ-secretase inhibitor 11-treated APP/PS1 mice travelled significantly longer time and distance than vehicle-treated mice (Fig. 7E, F), suggesting a substantial mitigation of impaired cognitive function. We then performed staining of 6E10 immunohistochemically (Fig. 7G). Quantification of the average percentage of area occupied by 6E10-positive stain showed that treatment with δ-secretase inhibitor 11 significantly reduced deposition of Aβ plaque in the hippocampus and cortex (Fig. 7H).

Above examination on APP/PS1 mice revealed that elevation of AEP is an early sign of AD pathology that precedes formation of senile plaques. The next step was to use this imaging probe to measure AEP activity in the brain of APP/PS1 mice.

### Use of AuNPs-Cy5.5-A&C to detect brain AEP activity of APP/PS1 mice

**Fig. 8:**
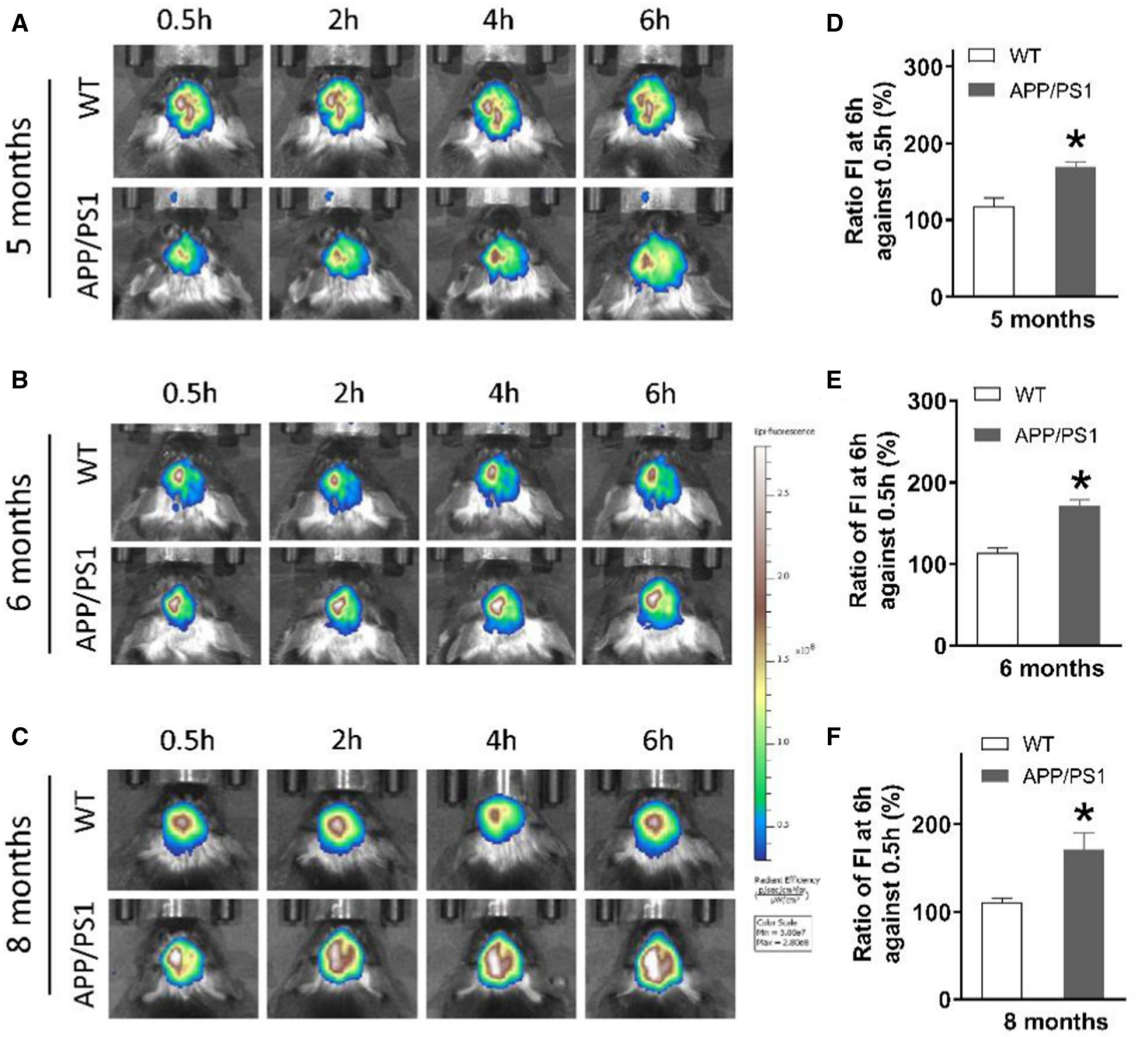
Imaging of AuNPs-Cy5.5-A&C in brains of the APP/PS1 and age-matched WT mice. **A**–**C** Two group of mice at 5, 6, and 8 months of age, imaging of the brain was performed at 0.5, 2, 4 and 6 h post intracerebroventricular injection of AuNPs-Cy5.5-A&C. **D**–**F** Ratio: the fluorescence intensity (FI) at 6 h divided by FI at 0.5 h post injection. *P < 0.05, comparison between APP/PS1 and age-mated WT mice, Student’s *t*-test. n = 5 mice per group

The enzymatic activity of brain AEP was up-regulated in 5 to 8-month old APP/PS1 mice in comparison with WT mice. Herein, we used AuNPs-Cy5.5-A&C to detect AEP activity in the brain of AD model mice. The BBB breakdown of APP/PS1 mice was not severe as that in TBI mice [33]; and this AEP probe might not be able to permeate into brain tissue. We injected AuNPs-Cy5.5-A&C into the left lateral ventricle of anesthetized mice to let these probes diffuse within the cerebrospinal fluid (CSF) circulation, then performed live animal imaging at 0.5, 2, 4 and 6 h after injection. Examination of 5, 6 and 8-month old APP/PS1 mice showed that fluorescence of the AEP probe gradually increased over the 6-h imaging course; however, there was no fluorescence increase in age-mated WT mice (Fig. 8A–C). We employed the ratio of fluorescence intensity (FI) at 6 h to FI at 0.5 h to evaluate the extent of fluorescence enhancement. Statistical results demonstrated that the fluorescence intensity of AEP imaging was significantly enhanced in the brain of APP/PS1 mice in comparison with age-mated WT mice (Fig. 8D–F). So far, this AEP imaging probe enabled us to monitor up-regulated brain AEP activity, which is an early marker of AD pathology that precedes formation of Aβ plaque and cognitive impairment.

## Discussion

The present study demonstrated, for the first time, that up-regulated AEP in the brain is a promising marker for predicting AD and this early “signature” can be detected by using brain imaging analysis. We revealed that AEP activity was enhanced in brain tissue of AD model mice at an early age and elevation of AEP preceded formation of Aβ plaque and cognition injury. Pharmacological inhibition of AEP reduced Aβ production and mitigated AD-like symptom. A parallel study performed in our lab also identified the involvement of AEP in brain ageing and AD-like pathogenesis of a senescence-accelerated mouse prone 8 mice [34]. These are in line with many studies that have reported the critical role of AEP in AD onset and progression of 5xFAD, 3xTg-AD and P301S tau transgenic mice [11–14, 16].

Nevertheless, to establish brain AEP as a new AD biomarker is not without any concern. AEP is also up-regulated and involved in progression of Parkinson’s disease (PD), TBI, and glioblastoma. In human brains with PD, activated AEP cleaves human α-synuclein to make them aggregate in Lewy bodies [35, 36]. Study of TBI documents that AEP expression and activity was elevated in associated brain region [15]. In human glioblastoma, AEP was found highly expressed and associated with poor prognosis [18]. To guarantee the specificity of AEP for AD diagnosis, other brain diseases should be distinguished. It is well known that affected brain region in AD is different from PD, which is featured by the degeneration of substantia nigra (SN) dopaminergic neurons and their projections into the striatum. PD can be specifically diagnosed with the selective dopaminergic radioligand-based PET imaging or magnetic resonance (MR) imaging of the SN projections [37, 38]. An accurate diagnosis of glioblastoma is through neurological exam of specific symptoms, magnetic resonance imaging (MRI) and computerized tomography (CT) scanning, and a brain biopsy [39]. TBI or stroke can be readily diagnosed by health history, symptoms, physical examination and CT scan. So, combined usage of physical exams and radiology approaches enables the differential diagnosis of brain diseases other than AD, allowing AEP to be developed into an AD biomarker.

The imaging probe we here used was constructed from the biocompatible particles AuNPs, which can be taken up by a cell and act as intracellular probes [40]. This AEP-responsive probe displayed a general applicability in various cell lines and the florescence intensity of AEP imaging correlated well with their AEP activity. This capability of AuNPs-Cy5.5-A&C enabled us to detect brain AEP activity in live animals which suffer from TBI and AD. In the model of TBI, there was elevated brain AEP and being disrupted BBB, which allowed AuNPs-Cy5.5-A&C to enter brain tissue after intravenous injection and to be activated by AEP. Therefore, we observed strong fluorescence of the probe that represents increased AEP activity. In the brain of sham control mice, there was no remarkable fluorescent because the imaging probe was not activated. As we know, brain AEP did not increase in sham control mice and the BBB wasn't as damaged as that in TBI. The present imaging probe AuNPs-Cy5.5-A&C might not be able to cross the BBB. This limitation temporarily hampered its translational application and needs to be fixed through enhancing its BBB permeability in future work. Upon the 5-month old and older APP/PS1 mice, we injected AuNPs-Cy5.5-A&C into the lateral ventricle of their brains because the BBB was not damaged as that in TBI model [33, 41]. We found that live brain imaging with AuNPs-Cy5.5-A&C truly reflected elevated AEP level at the early disease stage of AD mice (5 months old). Until the present work was accomplished, there were no AEP imaging studies reported in the field of neurodegenerative diseases. Previous imaging probes were primarily applied to tumors to explore the role of AEP in tumor progression and metastasis [42, 43]. To the best of our knowledge, we here showed for the first time that up-regulated AEP in AD brain can be monitored by live-animal imaging analysis.

## Conclusions

AD is a progressive condition and often undergoes a 15–20 years of asymptomatic pathology stage before dementia emerges [44]. We here showed that age-associated activation of brain AEP represents the early pathological stage of AD and AEP measurement by an imaging analysis could become a new AD biomarker. In addition, our present and previous studies have proved that pharmacological inhibition of AEP was an effective intervention to impede AD-like pathological progression. It is expected that the translational application of brain AEP imaging would be not only for early diagnosis of AD, but also for monitoring efficacy of drugs that target AEP as a treatment.

## Supplementary Information

**Additional file 1: Figure S1.** AEP-triggered fluorescence enhancement of AuNPs-Cy5.5-A&C. In pH 5.0 HEPES buffer, AuNPs-Cy5.5-A&C was incubated without or with AEP (1 mg ml^−1^) for 12 h. 20 μM Atorvastatin (Ato) was used to inhibit AEP. AuNPs-Cy5.5-AK or AuNPs-Cy5.5-CABT alone was also incubated in the same buffer. Fluorescent intensity was measured by a microplate reader. **P < 0.01, AEP treated group compared with other groups; one-way ANOVA, n = 3 independent experiments. **Figure S2.** Whole body fluorescence imaging of TBI mice. We have shaved hair on the head but kept the body hair intact when imaging mice. **Figure S3.** Spatial learning and memory of age-mated APP/PS1 and WT mice at 5, 6, and 8 months of age. **(A-B)** The latency to the escape platform and swimming speed of 5–6-month old mice over the 5-day acquisition training. **(C-D)** The percentage of time spent and distance travelled in the target quadrant in the hidden platform test (probe test), which was performed on day 6 after the acquisition period. No significant difference between APP/PS1 and WT mice at 5 and 6 months of age. *P* > 0.05, Student’s *t*-test, n = 8 mice per group**. (E–F)** The latency to the escape platform and swimming speed of 8-month old mice over the 5-day acquisition training. *P < 0.05, group × training day effect. n = 8 mice per group, two-way ANOVA. **(G-H)** The percentage of time spent and distance travelled in the target quadrant. *P < 0.05 and **P < 0.01, between groups, Student’s *t*-test, n = 8 per group. **Figure S4.** Aβ plaque deposition in the brain of age-mated APP/PS1 and WT mice at 5, 6, and 8 months of age. Staining of 6E10 (indicated by arrow head) in the hippocampus and cortex demonstrate that Aβ plaque began to appear at 6 months and expanded at 8 months of age. Scale bar = 250 μm. No significant Aβ plaque present in the brain of WT mice. **P < 0.01, Student’s *t*-test between groups; n = 5 mice in each group.

## Data Availability

The datasets used and/or analyzed in this study are available from the corresponding author on reasonable request.

## Abbreviations

AD: Alzheimer’s disease
ADD: AD dementia
MCI: Mild cognitive impairment
AEP: Asparaginyl endopeptidase
AuNPs: Gold nanoparticles
APP/PS1: APPswe/PS1dE9 transgenic mice
Aβ: β-Amyloid
CSF: Cerebrospinal fluid
APP: Amyloid precursor protein
HAuCl4: Chloroauric acid
AK: Alanine–alanine–asparagine–cysteine–lysine
CABT: 2-Cyano-6-aminobenzothiazole
DLS: Dynamic light scanning
TBI: Traumatic brain injury
CCI: Controlled cortical impact
Ato: Atorvastatin
BBB: Blood–brain-barrier
PD: Parkinson’s disease
MRI: Magnetic resonance imaging
CT: Computerized tomography
