# Supplementary material for: Imaging asparaginyl endopeptidase (AEP) in the live brain as a biomarker for Alzheimer’s disease
Source: J Nanobiotechnology. 2021 Aug 19;19:249. doi: 10.1186/s12951-021-00988-0 (PMC8375181; doi:10.1186/s12951-021-00988-0)
Supplement: Supplementary file 1 — Additional file 1: Figure S1. AEP-triggered fluorescence enhancement of AuNPs-Cy5.5-A&C. In pH 5.0 HEPES buffer, AuNPs-Cy5.5-A&C was incubated without or with AEP (1 mg ml−1) for 12 h. 20 μM Atorvastatin (Ato) was used to inhibit AEP. AuNPs-Cy5.5-AK or AuNPs-Cy5.5-CABT alone was also incubated in the same buffer. Fluorescent intensity was measured by a microplate reader. **P < 0.01, AEP treated group compared with other groups; one-way ANOVA, n = 3 independent experiments. Figure S2. Whole body fluorescence imaging of TBI mice. We have shaved hair on the head but kept the body hair intact when imaging mice. Figure S3. Spatial learning and memory of age-mated APP/PS1 and WT mice at 5, 6, and 8 months of age. (A-B) The latency to the escape platform and swimming speed of 5–6-month old mice over the 5-day acquisition training. (C-D) The percentage of time spent and distance travelled in the target quadrant in the hidden platform test (probe test), which was performed on day 6 after the acquisition period. No significant difference between APP/PS1 and WT mice at 5 and 6 months of age. P > 0.05, Student’s t-test, n = 8 mice per group. (E–F) The latency to the escape platform and swimming speed of 8-month old mice over the 5-day acquisition training. *P < 0.05, group × training day effect. n = 8 mice per group, two-way ANOVA. (G-H) The percentage of time spent and distance travelled in the target quadrant. *P < 0.05 and **P < 0.01, between groups, Student’s t-test, n = 8 per group. Figure S4. Aβ plaque deposition in the brain of age-mated APP/PS1 and WT mice at 5, 6, and 8 months of age. Staining of 6E10 (indicated by arrow head) in the hippocampus and cortex demonstrate that Aβ plaque began to appear at 6 months and expanded at 8 months of age. Scale bar = 250 μm. No significant Aβ plaque present in the brain of WT mice. **P < 0.01, Student’s t-test between groups; n = 5 mice in each group. [file 12951_2021_988_MOESM1_ESM.pdf]

Imaging Asparaginyl Endopeptidase (AEP) in the Live Brain as a Biomarker for Alzheimer's disease

Shan-Shan Wang[1 #], Zi-Kai Liu[1 #], Jing-Jing Liu[1 #], Qing Cheng[1], Yan-Xia Wang[1], Yan Liu[1], Wen-Wen Ni[1], Hong-Zhuan Chen[2] * and Mingke Song[1] *

Additional file: **Figure S1**

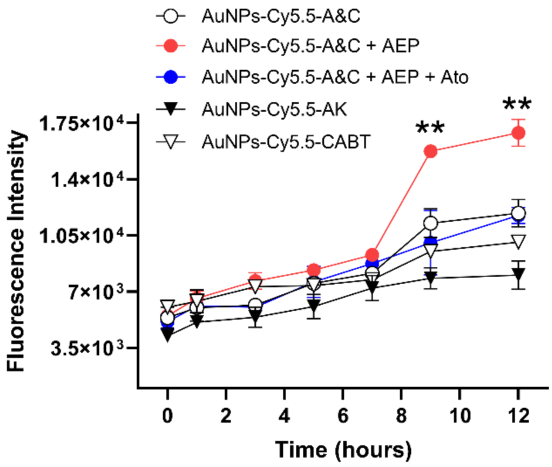

Additional file: **Figure S1.** AEP-triggered fluorescence enhancement of AuNPs-Cy5.5-A&C. In pH 5.0 HEPES buffer, AuNPs-Cy5.5-A&C was incubated without or with AEP (1 mg ml^{-1}) for 12 hours. 20 μM Atorvastatin (Ato) was used to inhibit AEP. AuNPs-Cy5.5-AK or AuNPs-Cy5.5-CABT alone was also incubated in the same buffer. Fluorescent intensity measured by a microplate reader. ***P* < 0.01, AEP treated group compared with other groups; one-way ANOVA, n = 3 independent experiments.

Additional file 1: **Figure S2**

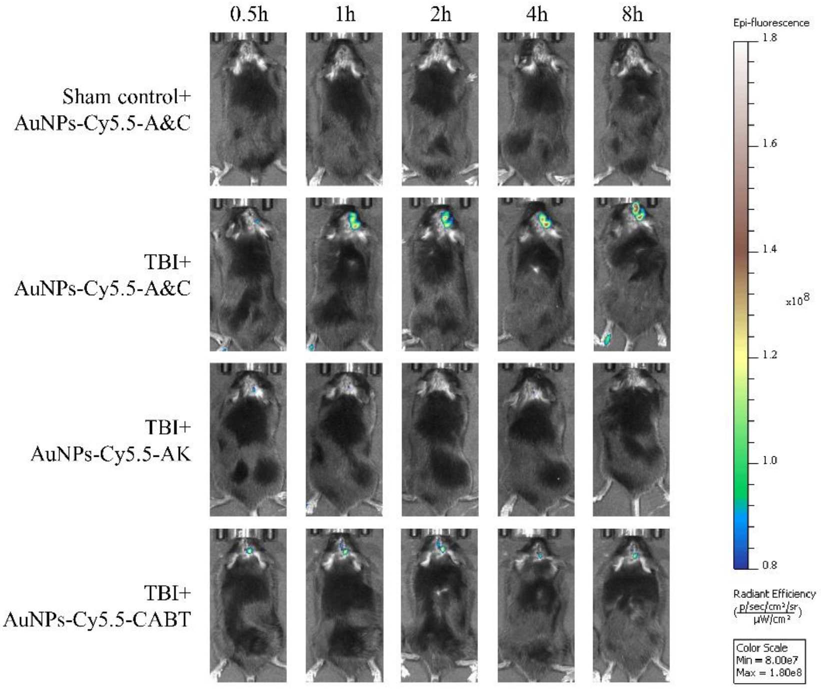

Additional file 1: **Figure S2.** Whole body fluorescence imaging of TBI mice. We have shaved hair on the head but kept the body hair intact when imaging mice.

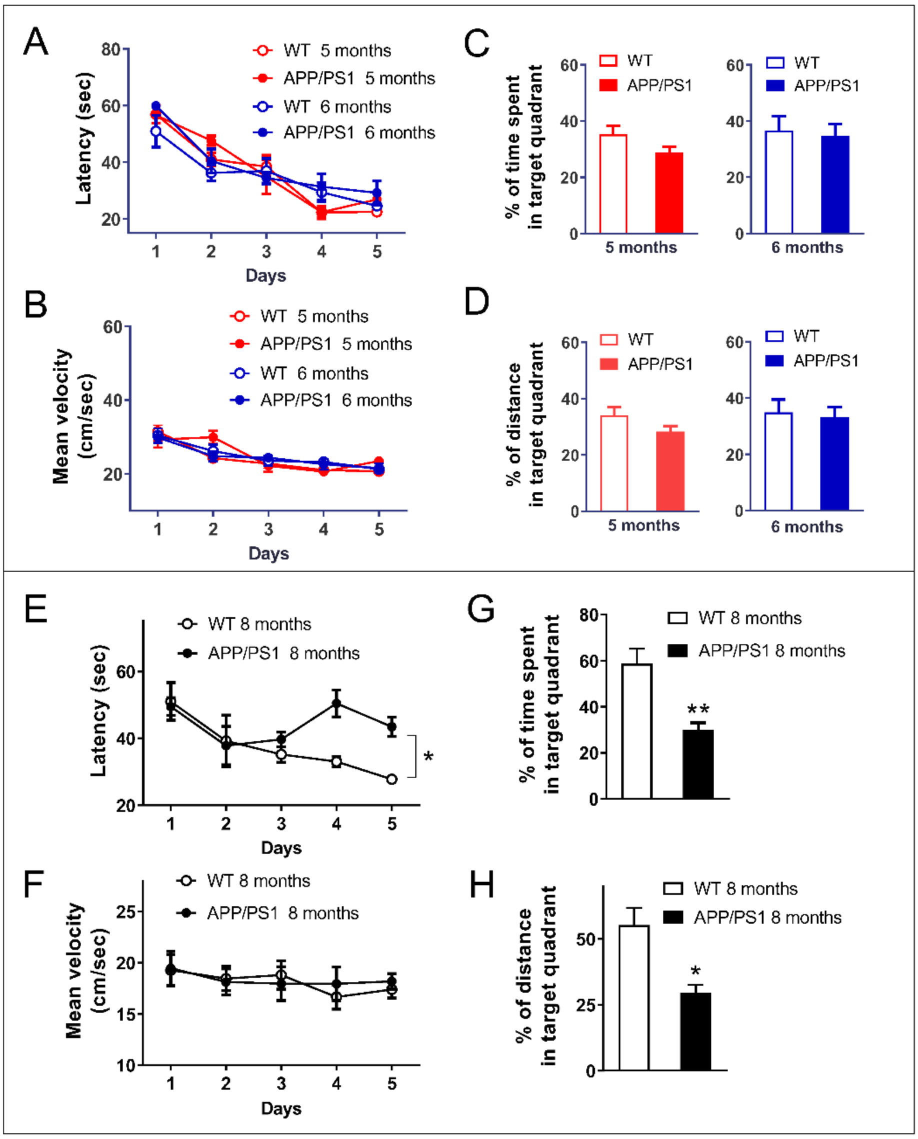

Additional file 1: **Figure S3.** Spatial learning and memory of age-mated APP/PS1 and WT mice at 5, 6, and 8 months of age. **(A-B)** The latency to the escape platform and swimming speed of 5-6-month old mice over the 5-day acquisition training. **(C-D)** The percentage of time spent and distance travelled in the target quadrant in the hidden platform test (probe test), which was performed on day 6 after the acquisition period. No significant difference between APP/PS1 and WT mice at 5 and 6 months of age. $P > 0.05$, Student's *t*-test, n = 8 mice per group. **(E-F)** The latency to the escape platform and swimming speed of 8-month old mice over the 5-day acquisition training. *$P < 0.05$, group × training day effect. n = 8 mice per group, two-way

ANOVA. **(G-H)** The percentage of time spent and distance travelled in the target quadrant. **P* < 0.05 and ***P* < 0.01, between groups, Student's *t*-test, n = 8 per group.

Additional file 1: **Figure S4.**

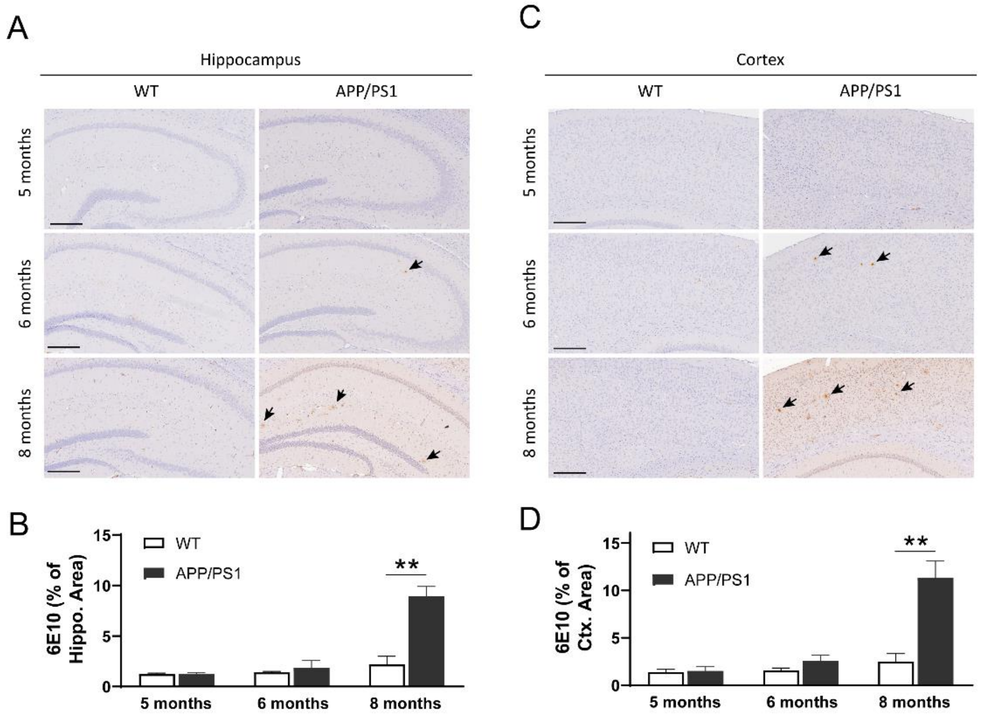

Additional file 1: **Figure S4.** Aβ plaque deposition in the brain of age-mated APP/PS1 and WT mice at 5, 6, and 8 months of age. Staining of 6E10 (indicated by arrow head) in the hippocampus and cortex demonstrate that Aβ plaque began to appear at 6 months and expanded at 8 months of age. Scale bar = 250 μm. No significant Aβ plaque present in the brain of WT mice. ***P* < 0.01, Student's *t*-test between groups; n = 5 mice in each group.
